# SARS-CoV-2 Detection in International Travelers Through Wastewater-Based Epidemiology at the Kigali International Airport: Genomic Surveillance Study

**DOI:** 10.2196/71104

**Published:** 2025-11-27

**Authors:** Rwagasore Edson, Robert Rutayisire, Ziad El-Khatib, Olivier Nsekuye, Hugues Valois Mucunguzi, Raissa Muvunyi, Esperance Umumararungu, Eric Remera

**Affiliations:** 1 Rwanda Biomedical Center, KG 644 St, Kimihurura, Kigali, P.O. Box 7162, Rwanda, 250 788 599 240; 2 Department of Global Public Health, Karolinska Institutet, Stockholm, Sweden

**Keywords:** COVID-19 pandemic, SARS-CoV-2, community-based surveillance, building health systems resilience, low-income countries, pandemic preparedness

## Abstract

**Background:**

Traditional infectious disease surveillance systems face significant limitations, including delayed detection, underreporting of asymptomatic cases, and inequitable health care access. Wastewater-based epidemiology (WBE), enhanced with genomic analysis, offers a noninvasive and cost-effective alternative for early pathogen detection and variant characterization, particularly valuable for monitoring international disease transmission.

**Objective:**

This study aimed to implement and evaluate a genomics-enhanced WBE surveillance system for detecting and characterizing SARS-CoV-2 variants among international travelers at the Kigali International Airport, Rwanda, and to assess its potential as an early warning system for pandemic preparedness.

**Methods:**

Between May and December 2023, we collected wastewater samples from international flights arriving at the Kigali International Airport under Rwanda’s National One Health strategy. Molecular detection was performed using polymerase chain reaction (PCR) assays, followed by whole-genome sequencing of positive samples. Bioinformatics analysis included quality assessment with Nanoplot (version 1.41.6), genome mapping using minimap2 (version 2.26), and lineage identification using the Freyja tool (version 1.4.5). Spatial and temporal analyses were used to identify transmission patterns and variant origins.

**Results:**

Of 630 wastewater samples collected from flights originating from 9 countries, 603 were successfully processed, with 21% (132/617) testing positive for SARS-CoV-2. Whole-genome sequencing was conducted on 33 samples, yielding an average viral sequence depth of 1250 reads with 92% genome coverage (range 78%‐97%). Genomic analysis identified 7 SARS-CoV-2 variants, including Omicron subvariants XBB.1.5, XBB.1.16.6 (eg, 5.1), GE.1, and FE.1.1.1. Notably, 70% (23/33) of sequenced samples could not be assigned to existing lineages, suggesting potential novel variants. Most samples came from Qatar (21.4%, 135/1630), the United Arab Emirates (19.5%, 123/1630), and the United Kingdom (19.4%, 122/1630). Positive samples were detected from 11 countries, with variants frequently found in flights from the United Kingdom, France, Belgium, Kenya, Tanzania, and South Africa. Sample collection capacity increased from 6 in week 1 to 33 by week 27. SARS-CoV-2 positivity rates showed seasonal variation, with a marked decline in June-July 2023.

**Conclusions:**

Genomics-enhanced WBE demonstrated a high sensitivity for the early detection of SARS-CoV-2 variants among international travelers, including potential novel variants undetectable through traditional surveillance. Its noninvasive and cost-effective nature, combined with the ability to generate population-level epidemiological insights, makes it particularly suitable for resource-limited settings. This approach supports Rwanda’s National One Health strategy and offers a scalable model for advancing global health security in Sub-Saharan Africa through innovative surveillance tools.

## Introduction

The emergence of the severe acute respiratory syndrome coronavirus 2 (SARS-CoV-2), which led to the global COVID-19 pandemic, represents one of the most significant global health crises of the 21st century. The pandemic challenged health systems worldwide in their preparedness, readiness, and capacity to respond effectively to a crisis of such magnitude [1]. More importantly, the rapid spread of the pandemic exposed delays and limitations in existing surveillance systems, underscoring the urgent need to restructure and incorporate advanced tools and novel strategies to improve public health surveillance and response systems [2-4].

The conventional approach to disease surveillance typically relies on clinical reporting and individual testing, which faces significant limitations including delayed detection, underreporting due to asymptomatic cases, and inequitable access to health care services. These limitations became particularly evident during the COVID-19 pandemic, highlighting the need for complementary surveillance methods that can overcome these challenges.

As part of global efforts to enhance epidemiological surveillance, the approach of wastewater-based epidemiology (WBE) has emerged. Therefore, WBE is the current science of analyzing wastewater to harvest the biological and chemical indicators of community health and risks. By detecting and quantifying biomarkers such as chemicals, pharmaceuticals, pathogens, and antimicrobial resistance genes in wastewater, WBE offers a powerful surveillance tool [5,6].

Recent advances in WBE have demonstrated its effectiveness in monitoring various pathogens beyond SARS-CoV-2, including influenza viruses, norovirus, and antimicrobial resistance genes. WBE can detect pathogen signals 5‐7 days before clinical cases are reported, providing crucial early warning for public health interventions. Furthermore, the integration of WBE with digital disease surveillance and artificial intelligence has shown promise in enhancing the sensitivity and specificity of outbreak detection systems. Additionally, WBE can be expanded to include the surveillance of disease vectors with aquatic life stages, such as mosquitoes and snails, through the detection and characterization of environmental DNA [7-9].

The central premise of WBE is that wastewater provides a noninvasive method to collect pooled community samples containing epidemiological data, which is critical for monitoring public health and associated risks within a given area. Furthermore, combining WBE with advanced diagnostic tools, such as genomics and molecular diagnostics, enhances surveillance systems by enabling the early detection of locally circulating pathogens. This integrated approach provides an effective early warning system for disease outbreaks [5]. Molecular and genomic sequencing tools have proven sensitive enough to detect SARS-CoV-2 in sewage and wastewater, validating the utility of WBE in pandemic response efforts [10,11].

In response to the COVID-19 pandemic, Rwanda invested in strengthening its surveillance systems following the World Health Organization’s (WHO) declaration of COVID-19 as a public health emergency of international concern in early 2020. Rwanda monitored the spread of COVID-19 through molecular detection and contact tracing, deploying locally adapted measures to control transmission. According to the WHO, Rwanda reported 133,194 confirmed cases and 1468 deaths attributable to COVID-19 [12].

Like many other low-income countries, Rwanda faces significant public health challenges, including recurrent infectious disease outbreaks, antimicrobial resistance, and the impacts of climate change. The early detection of health threats is critical to enabling rapid responses that limit further spread and mitigate negative outcomes. However, traditional surveillance approaches are often limited by the data they can collect and provide, resulting in reduced effectiveness in informing policymakers and guiding public health interventions. This highlights the urgent need to adopt innovative approaches and robust tools such as WBE, which can generate real-time, community-based epidemiological data.

To address these challenges, Rwanda is investing in implementing a genomics-enhanced WBE surveillance system as part of its National One Health strategy for early preparedness and response [13,14]. This strategy integrates genomic surveillance with public health response, universal health coverage, and the use of advanced technologies, such as drones, to improve animal, human, and environmental health services. In this study, we presented the integration of WBE and genomic surveillance in Kigali, Rwanda, to monitor the dynamics of infectious diseases, with a focus on COVID-19 among international travelers.

## Methods

### Overview

**Figure 1.**
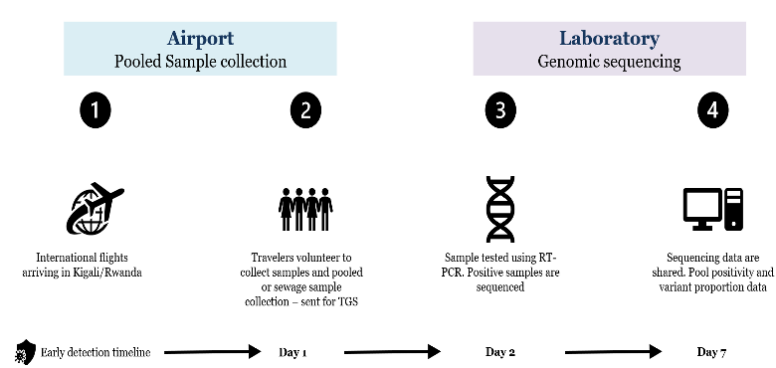
Implementation of the genomics-enhanced wastewater-based epidemiology (WBE) surveillance system for SARS-CoV-2 detection at the Kigali International Airport, Rwanda (May-December 2023).

The Rwanda Pathogen Monitoring program, based on WBE, was launched in May 2023. This program implemented WBE at the Kigali International Airport, the main international airport in Rwanda, to detect and monitor variants of severe acute respiratory SARS-CoV-2 among international travelers. Wastewater monitoring for COVID-19 on flights involved the collection and preservation of sewage samples from aircraft wastewater systems. These samples were analyzed using molecular and genomics tools to detect SARS-CoV-2. The program was complemented by collecting individual biological samples from international travelers who voluntarily participated in the study. Genomic analysis was performed on these individual samples, and the results were compared to those obtained from wastewater samples to assess the sensitivity and capacity of WBE for monitoring cross-border transmission of SARS-CoV-2. Figure 1 illustrates the structure of the genomics-enhanced WBE surveillance system implemented in the Kigali International Airport for monitoring the dynamics of SARS-CoV-2 among international passengers entering Rwanda.

### Aircraft Wastewater Sampling

The WBE approach was implemented following a well-established protocol developed by the Rwanda Biomedical Center (RBC). Briefly, aircraft wastewater sampling involved the collection and analysis of wastewater from grounded aircraft to monitor the presence of pathogens. The process began by accessing the aircraft’s wastewater system and connecting it to the waste valve of the aircraft lavatory system. Samples were collected using aseptic techniques to prevent contamination, using sterilized containers and tools to ensure the representativeness of the wastewater in the system. Following collection, the samples were transported to the Rwanda National Reference Laboratory under strict conditions to maintain appropriate storage temperatures and preserve sample integrity. At the laboratory, the samples underwent molecular and genomic analysis to detect circulating variants of interest. Results were interpreted in alignment with established protocols to ensure consistency and reliability.

### Detection of SARS-CoV-2

Molecular analysis using polymerase chain reaction (PCR) assays was used to detect and quantify SARS-CoV-2 viral loads. All samples testing positive for SARS-CoV-2 via PCR were further subsequently subjected to genomic sequencing to identify viral lineages. A genomic surveillance pipeline was developed and implemented to detect the virus in travelers and wastewater samples and to characterize circulating variants. This pipeline maintained established links between passengers and flights to allow for validation of findings.

### Data Analysis of Raw Reads

Bioinformatics tools were used for data analysis. Amplicon reads were assessed for quality using Nanoplot (version 1.41.6). Reads were then mapped to the SARS-CoV-2 reference genome (GenBank accession no NC_045512) using minimap2 (version 2.26). Subsequently, genome indexing was performed to produce sorted and indexed BAM files using SAMtools (version 1.17). The Freyja tool (version 1.4.5) was used to capture the abundance of variants lineages in wastewater samples.

### Ethical Considerations

This study was conducted within the public health surveillance mandate of the RBC and was deemed exempt from ethical review, as it involved only the analysis of wastewater samples collected from aircrafts, with no human subject involvement whatsoever. No personal data were collected, processed, or stored at any point during this research. The wastewater sampling was performed as part of routine public health surveillance activities, in accordance with international regulations for disease monitoring at points of entry. All collected samples were completely anonymized, with only flight origin data retained for epidemiological analysis. The study adhered to established biosafety protocols for handling wastewater samples potentially containing infectious materials.

## Results

### Overview

**Figure 2.**
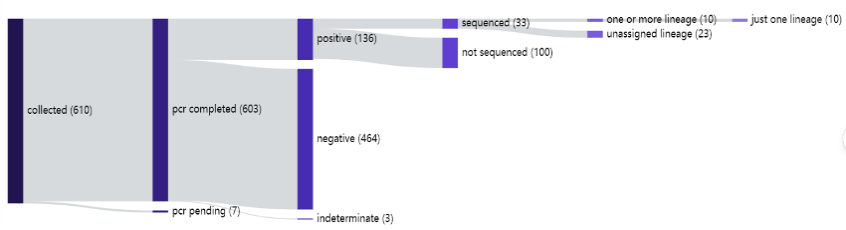
Surveillance algorithm for COVID-19 detection in wastewater samples collected from international flights arriving at the Kigali International Airport, Rwanda (May-December 2023).

A total of 630 wastewater samples were collected, with 603 being successfully tested. Among these, 21% (132/617) tested positive for SARS-CoV-2. Whole-genome sequencing was performed on 33 of the positive samples. Figure 2 outlines the surveillance algorithm for genomics-enhanced WBE used to monitor SARS-CoV-2 among international travelers arriving in Kigali.

### Temporal Analysis of the Genomics-Enhanced WBE Surveillance for COVID-19 Among International Flights Arriving in Rwanda

**Figure 3.**
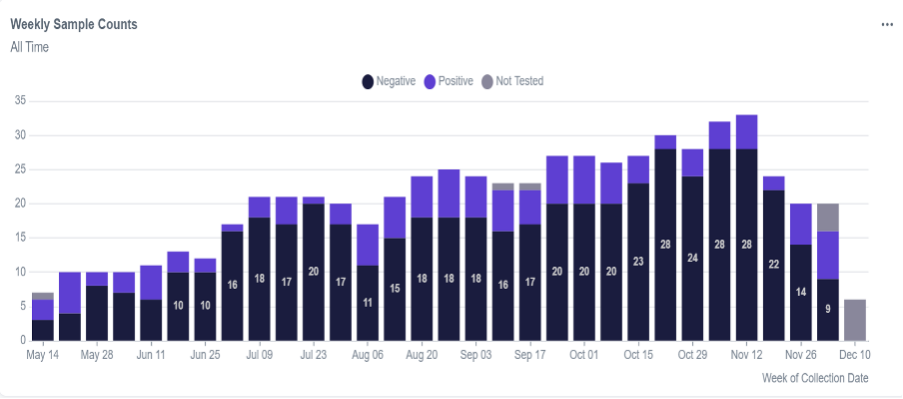
Temporal trends of SARS-COV-2 testing and positivity rate in wastewater samples from international flights arriving at the Kigali International Airport between May 14 and December 10, 2023.

The capacity of the surveillance system improved over time, as indicated by a significant increase in the weekly number of wastewater samples collected. Sample collection increased from 6 samples in the first week (May 14‐19, 2023) to 33 samples in week 27 (November 12‐18, 2023; Figure 3). However, a major decline in the SARS-CoV-2 positivity rate was observed between June and July, with only a single positive sample recorded during the period of 26 June to 23 July. Figure 3 illustrates biweekly changes in wastewater sample collection and seasonal variations in SARS-CoV-2 positivity rates among commercial flights arriving at the Kigali International Airport.

### Spatial Analysis of the Genomics-Enhanced WBE Surveillance for COVID-19 Among International Flights Arriving in Rwanda

**Figure 4.**
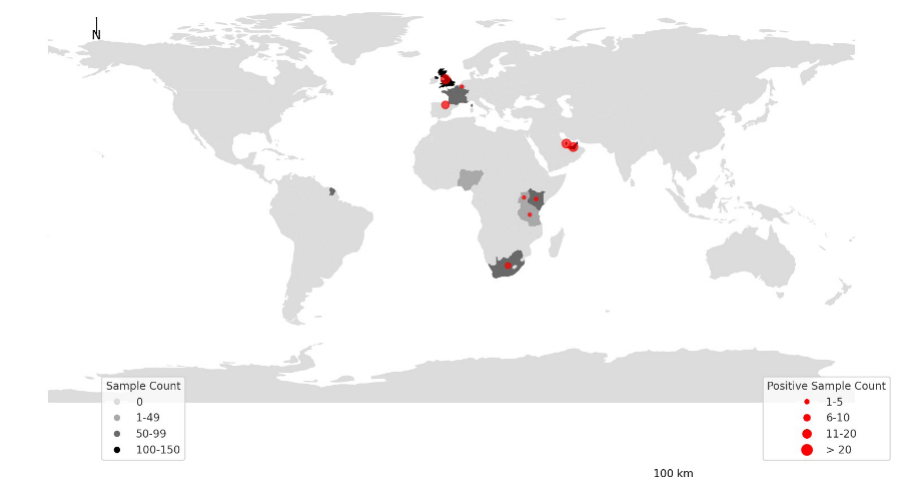
World map highlights the distribution of the collected and COVID-19 positive wastewater samples by the country of origin that was detected by the genomics-enhanced wastewater-based epidemiology surveillance system in Rwanda in 2023.

The 630 wastewater samples were collected from flights arriving from 9 countries. The majority of samples obtained from flights arriving from Qatar (21.4%, 135/630) followed by the United Arab Emirates (19.5%, 123/630) and the United Kingdom (19.4%, 122/630). Positive samples were identified from flights arriving from 11 countries, including the United Kingdom, France, Belgium, Kenya, Tanzania, the United Arab Emirates, Qatar, Tanzania, South Africa, and Uganda (Figure 4).

### Lineage Description

**Figure 5.**
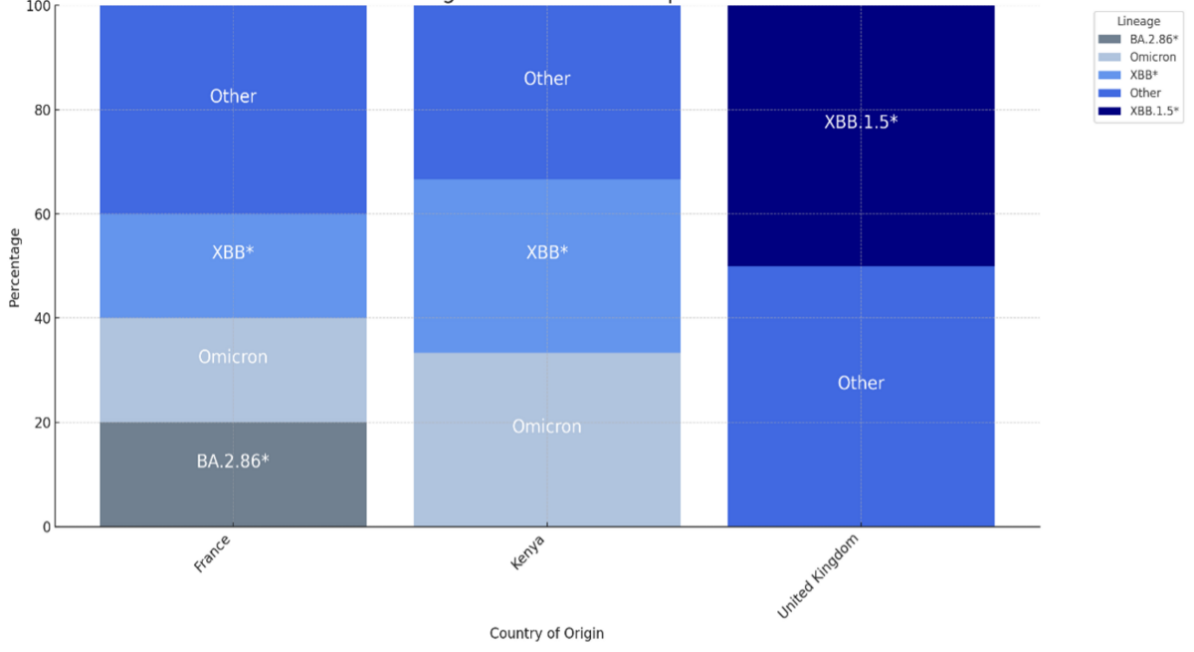
The distribution of SARS-CoV-2 lineages distributed per the country of origin.

Of the 33 COVID-19 positive wastewater samples sequenced, 23 could not be assigned to any known lineages. Genomic analysis of the remaining 10 samples revealed a high diversity of currently circulating internationally variants of SARS-CoV-2. Seven distinct Omicron subvariants were detected, including XBB.1.5, XBB.1.16.6 (eg, 5.1), GE.1, and FE.1.1.1. This included both recombinant variants such as XBB.1.5 and XBB.1.16.6, as well as subvariants from established Omicron lineages, including BA.5 (eg, 5.1), BA.2 (eg, GE.1) and BA.1 (eg, FE.1.1.1). Notably, XBB, XBB.1.5, BA.2.86, and other Omicron subvariants were the most prevalent and were primarily detected in wastewater from flights arriving from France, Kenya, and the United Kingdom (Figure 5).

The high proportion (70%, 23/33) of unassigned sequences suggests the potential emergence of novel variants. However, further investigation is required to understand the evolution and dynamics of these variants.

## Discussion

### Principal Findings and Comparison With Previous Works

In this study, we presented a proof-of-concept study that emphasized the potential of genomics-enhanced WBE surveillance in monitoring the dynamics of the COVID-19 pandemic across countries via international flights and in detecting the spread of SARS-CoV-2 variants. The findings demonstrate the feasibility and effectiveness of WBE in the early detention of SARS-CoV-2 and circulating variants, highlighting its value in contributing to global heath security and pandemic preparedness. The diversity of SARS-CoV-2 origins detected underscores the utility of genomics-enhanced WBE for infectious disease surveillance.

Genomics-enhanced WBE offers an opportunity to track and monitor foodborne and waterborne diseases at the community level. This innovative approach aligns with Rwanda’s National One Health strategy, which aims to improve human, animal, and environmental health through an integrated, transdisciplinary approach and multisectoral collaboration [15-17]. Additionally, it holds potential for advancing the control of neglected tropical diseases and reducing their socioeconomic burden [15]. Findings of this analysis demonstrate the feasibility and effectiveness of the use of WBE for the early detection of SARS-CoV-2 and the circulating variants. Furthermore, it highlights the diversity of origins in the source of SARS-CoV-2, contributing to global health security and pandemic preparedness.

The high proportion (70%, 23/33) of unassigned sequences suggests the potential emergence of novel variants. However, further investigation is required to understand the evolution and dynamics of these variants. This finding underscores the significant potential of WBE as an early warning system for detecting novel pathogen variants before they are identified through clinical surveillance. The ability to detect unassigned SARS-CoV-2 sequences through airport wastewater monitoring demonstrates how this approach could serve as a critical first line of defense in identifying emerging pandemic threats at their earliest stages, potentially providing weeks of additional preparation time for public health responses before widespread community transmission occurs.

This project has built the national capacity in Rwanda, a low-income country, to implement an innovative surveillance system that successfully detected new SARS-CoV-2 variants in Rwanda for the first time. The robustness and cost-effectiveness of this system highlight its potential for scaling. The integration of this surveillance system with immediate response measures—such as community engagement, public awareness campaigns on respiratory disease prevention, and COVID-19 vaccination efforts—has strengthened Rwanda’s pandemic preparedness. These measures not only protect Rwandans but also safeguard international travelers by reducing the risk of disease spread or introduction of new variants into previously unaffected regions.

The implementation of genomics-enhanced WBE in Rwanda serves as a model for enhancing global health security in Sub-Saharan Africa. By building local capacity and adopting innovative approaches, Rwanda demonstrates how to effectively implement the One Health Strategy for improved health outcomes [18]. Expanding this cost-effective strategy both locally and internationally could help establish an early warning and response system to save lives and resources [4,19]. Such systems could be instrumental in densely populated areas with limited access to health care, particularly for monitoring infectious diseases with a high epidemic potential [5].

The emergence of WBE has transformed public health surveillance by providing real-time data on community health through the analysis of biomarkers in wastewater. This noninvasive approach reduces health care access challenges and socioeconomic barriers; public health systems often face challenges such as underreporting due to stigmatization or limited health care access, particularly in low-income settings. In contrast, WBE bypasses these bottlenecks by offering an unbiased snapshot of community health. Moreover, genomics-enhanced WBE aligns with the International Health Regulations [20] by facilitating cross-border data sharing and collaboration, improving global preparedness for future pandemics [21-23].

In this study, WBE not only identified new SARS-CoV-2 variants but also pinpointed their countries of origin (Figure 5). This highlights the importance of regionally coordinated data-sharing strategies to strengthen cross-border cooperation and enhance pandemic preparedness [21-23]. Integrating WBE with Rwanda’s existing digital disease surveillance infrastructure would further maximize its impact. Linking wastewater findings to individual diagnostic syndromic surveillance data could validate signals and reveal broader transmission patterns. Additionally, a digitized WBE system incorporating big data analysis and artificial intelligence can automate sample processing and interpretation, rapidly uncovering health threats and trends.

Beyond infectious diseases, WBE holds promise for monitoring antimicrobial resistance, substance use trends, disease vector composition, and the unregulated use of pesticides and fertilizers. This could inform strategies to combat antibiotics, insecticides and pesticides resistance, as well as addiction-related harms while guiding vector control efforts. When applied ethically, WBE generates actionable and equitable health insights unattainable through individual testing alone.

Focusing on the Kigali International Airport underscores Rwanda’s strategic approach to infectious disease surveillance. As a primary point of entry, the airport allows for the swift detection and mitigation of potential threats. The detection of 7 different Omicron subvariants in wastewater samples from international flights demonstrates the technique’s sensitivity and ability to identify diverse viral strains. This not only supports domestic public health response strategies but also contributes to global knowledge on viral evolution and transmission.

Using WBE for SARS-CoV-2 detection at airports has numerous advantages. It enables early detection, even before travelers develop symptoms or widespread community transmission occurs. Given that a significant proportion of asymptomatic travelers may carry SARS-CoV-2 [24,25]. WBE provides a cost-effective and noninvasive alternative to individual testing [26]. Unlike health declarations and temperature checks, which rely on passenger honesty and symptomatic presentations, WBE offers an objective measure of viral prevalence. Additionally, its nonintrusive nature respects passenger privacy (because data are collected in an anonymous fashion), making it an ethical surveillance tool. As new variants of SARS-CoV-2 emerge, WBE can detect shifts in viral strains within wastewater, enabling timely responses to potential outbreaks [27].

### Limitations

A key limitation of this study is the relatively small number of samples (n=33) that underwent full genomic sequencing, which may not capture the complete diversity of SARS-CoV-2 variants entering Rwanda through air travel. Additionally, while we collected complementary individual samples from travelers, we were unable to perform a direct comparison between individual and wastewater results for all flights due to logistical constraints and varying participation rates.

Furthermore, our sequencing data varied in quality across samples, with genome coverage ranging from 78% to 97% (median 92%, IQR 87%–95%) and depth of coverage varying from 850 to 1650 reads (mean [SD] 1250 [200] reads). The sequenced samples were not evenly distributed across the study period, with more samples sequenced during July to September and November, which may have affected our ability to detect temporal trends in variant circulation. Future studies should aim for a more consistent temporal distribution of sequencing to better track variant evolution over time.

However, the expansion of genomics-enhanced WBE in Rwanda faces funding challenges that could hinder scaling efforts. Sustainable financing models, such as donor-implementer partnerships and public-private collaborations, are essential for institutionalizing the system. Capacity building through knowledge training and knowledge exchange will foster self-sufficiency. Addressing these challenges through locally tailored strategies will ensure the long-term success of WBE. We call upon all health stakeholders to mobilize resources and strengthen collaboration to support pandemic preparedness through investments in genomics-enhanced WBE surveillance, harnessing wastewater surveillance to improve global health.

### Conclusions

The implementation of WBE for SARS-CoV-2 surveillance at the Kigali International Airport showed a strong potential of wastewater surveillance for public health in Rwanda. The system’s sensitivity in detecting COVID-19 prevalence and emerging variants among international travelers underscores WBE’s capability for real-time monitoring of public health threats. Expanding wastewater monitoring to communities across Rwanda could provide invaluable insights into population health, particularly among marginalized and underserved groups.

To fully realize the potential of WBE nationwide, further research and multi-stakeholder collaboration is essential. Optimizing decentralized sampling strategies and integrating WBE findings with clinical diagnostics and digital disease surveillance systems would significantly enhance its impact. Sustainable financing models and capacity-building initiatives are critical to ensuring that WBE becomes a cornerstone of Rwanda’s public health infrastructure.

Pilot WBE programs at other points of entry, densely populated urban areas, public institutions, and isolated rural communities could provide important insights for scaling up the approach. Initially, with additional support, Rwanda could prioritize the use of genomics-enhanced WBE to detect high-risk pathogens of global concern, such as polio virus, cholera, and typhoid. Over time, this system could be expanded to include broader disease surveillance, antimicrobial resistance monitoring, and the tracking of biochemical markers.

## Abbreviations

PCR: polymerase chain reaction
RBC: Rwanda Biomedical Center
SARS-CoV-2: severe acute respiratory syndrome coronavirus 2
WBE: wastewater-based epidemiology
WHO: World Health Organization
